# Abstract of Proceedings of the Buffalo Medical Association

**Published:** 1874-10

**Authors:** E. R. Barnes

**Affiliations:** Secretary


ART. III.—*Abstract of the Proceedings of the Buffalo Medical Association,* Buffalo, September 1st, 1874. Reported by E. R. BARNES, M. D., Secretary.

Members present: The Vice-President, Dr. Gould, in the chair, and Drs. Samo, Harding, Lynde, Hauenstein, Miner, Cronyn, Brush and Shaw.

Reading of minutes of last meeting dispensed with.

On motion, Drs. W. W. Miner, H. R. Hopkins and D. E. Chace, were admitted to membership, and the application of Dr. Bartow was received.

No reports of committees.

Voluntary communications being in order, Dr. BARNES presented specimens of large condylomatous tumors, which he had removed by the galvano-cautery.

The patient, Mrs. A. W., aged 19, had been admitted to the Buffalo General Hospital, Aug. 8th ult., having then reached nearly the eighth month of pregnancy—for the purpose of receiving such treatment for these growths as might be deemed advisable before her confinement. The tumors then formed a large bulging mass of vegetations, presenting the appearance of a single tumor, filling the space between the nates, and extending from the end of the coccyx posteriorly to the commissure of the vulva anteriorly. The surface at this time was uneven or nodular, and of a brownish color from the incrustation of the discharge. By lifting the edges it could be seen that they extended beyond the base; a further examination showed that the greater portion of the tumor consisted of two parts attached, each, by a large pedicle to opposite surfaces of the nates.

The patient had first noticed these growths about three months before admission, which showed a rapid increase in size. There was no history or other evidence of syphilis, nor of other venereal disease.

It was decided to remove these excrescences at once, as they seemed to cause much annoyance and constitutional irritation, and might retard recovery. The woman was not strong, and the growths were very vascular. Hence it seemed to be a case peculiarly adapted for removal by the galvano-cautery, which would insure less hemmorrhage than the knife, less objectionable after processes than transfixion and ligation, and would act more quickly and, perhaps with less shock than écraseur.

Dr. B. accordingly purchased the cautery instrument, which has been offered for sale in Buffalo for some time past; but while awaiting a supply of platinum wire, on Aug. 12th, the patient miscarried, giving birth to a living and apparently healthy child.

The miscarriage was supposed to be due to the irritation caused by the tumors. In consequence of this occurrence, the operation was deferred.

The progress of the patient after confinement was unfavorable. The secretion of milk took place, but was rather scanty. The pulse varied between 110 and 120 per minute. The size of the tumors made it necessary to lie always upon the side. A compress wet with a weak solution of carbolic acid had been kept upon the growths, and had removed the incrustation. The surface was now of a dull red color, uneven, like a mass of large exuberant granulations, or like a cauliflower, and covered with an abundant, thin purulent discharge, having a strong fetid odor.

On Aug. 27, the patient had two severe chills, for which quinine was given, and they did not return on the following day or the next. But the pulse remained at 120, the countenance was pale, sallow and expressive of suffering, and there was now a continuous pain referred to the abdominal region, although pressure did not show great tenderness in that region. The vegetations had grown very rapidly, and were now larger by one-third, than when the patient was admitted. The patient was getting worse, and her general condition was such as to excite anxiety. Believing that the growths were a partial if not the sole cause of this condition, it was decided to remove them at once.

On Aug. 29, the patient was placed under the influence of chloroform, and the tumors were removed in the presence of quite a number of medical gentlemen, who were attracted no doubt by a curiosity to witness the working of a machine, which has not yet become practically, very generally familiar to the profession in this vicinity. The first attempt was to employ the cautery knife, and it failed, because of an accident which prevented the further descent of the plates into the battery fluid, and, perhaps, also because of the ignorance of the operator as to the proper method of using the knife. The blade was first brought to a white heat and then applied to the base of the tumor, into which it sank, hissing, until the blade was nearly covered; then it lost its white heat and its further progress was arrested. The abstraction of heat, from so large a surface kept in contact with the tissues, was too rapid, and the use of the knife was abandoned, not however without having derived some valuable hints from the failure.

A loop of platinum wire was then placed about the base of one of the tumors, and when the current from the battery was allowed to traverse it, very promptly severed the connection with the subjacent tissues. The process was repeated with the opposite mass. There was little bleeding, simply a slight oozing.

There remained now four or five small growths attached by smaller pedicles, in the space between the anus and the posterior commissure of the vulva, which might have been readily removed by the cautery knife. But they were removed by the scissors, and the removal was followed by copious hemmorrhage, which was arrested by the application of a porcelain point, heated to a white heat by the battery.

On the day after the operation the patient's pulse, which before had been 120, fell to 84, the symptoms of constitutional irritation disappeared, and she seems now, Sept. 1, to promise a speedy recovery.

Dr. Cronyn said that growths of the character presented, and occupying the same locality, at or near the orifices of canals lined with mucous membrane, were common, both in the male and female. They grow very rapidly and sometimes attain an enormous size. They are friable in structure, breaking up easily, and bleeding profusely. About fourteen years ago he removed one of great size by transfixing it with four needles, and firmly applying ligatures so as to produce strangulation. The galvano-cautery had been quite recently introduced in Europe, and had been used in most of the hospitals there, but was receeding in favor. In the case presented, the scissors would have more rapidly removed the tumors. The slough left by the cautery was troublesome. Mr. Bryant had reported cases operated on by the galvano-cautery, cases of fistula in ano, and hemmorrhoidal and other tumors. But on analysis it would be found that they could not be classed properly as operations by the galvano-cautery, as the tumors were cut out in the ordinary way, and the cautery applied afterwards. After mentioning some cases which had come under his observation, Dr. Cronyn said that the results after the use of the cautery, were no better than after the use of the ligature or écraseur. The cautery might act quickly when applied, but much time was required for the necessary preparations. The cautery was apt to remove tissue more deeply than was desirable.

These growths are generally of syphilitic origin.

Dr. HAUENSTEIN narrated a case where he had removed from the person of a woman, by the écraseur, a large lobulated tumor of the class under consideration. There was very little bleeding. He had a suspicion of venereal origin. Iodide of potassium was administered, and the tumor had not returned.

Dr. SHAW had never seen a case which was not connected with syphilis.

Dr. CRONYN said that the distinction between those growths which were of syphilitic and those which were of non-syphilitic origin, was their resemblance in the former case to a wart, and in the latter case to a sponge. Dr. C. did not think, when the loop of platinum wire was used in connection with the battery, that it acted by cauterization. The elastic bandage of Esmarch had been lauded for its usefulness in preventing hemmorrhage during operations. Now the best opinion is, it does this only to give rise to a greater hemmorrhage after its removal. The wire loop does not act by cauterization, but simply as an écraseur.

Dr. LYNDE thought that the wire used with the battery was too small to remove tumors by its tension. He understood that the depth of tissue removed was not more than from an eighth to a sixteenth of an inch, which was not more than would follow the use of the écraseur. As to the cause of these growths, either he had not been able to discriminate properly, or his experience had not embraced all of the varieties which exist, but he felt assured of the pre-existence of syphilis in every case which had come under his observation.

He had examined the battery with a view to its use for the removal of a tumor at the posterior part of the pharynx, but did not think it adapted for the purpose, and had removed the tumor by the écraseur.

Dr. MINER said that these growths are generally called syphilitic. If we mean that the patient has had syphilis, and that these excrescences arise from the action of the syphilitic virus, we will be frequently wrong. They occur sometimes in persons who have never had sexual connection. The discharge from a gonnorrhœa is the most frequent cause. That from non-infecting sores will also produce them. Anything which irritates the mucous membrane may cause their development. These growths are almost always venereal, not often syphilitic. Consequently he would not regard the existence of these growths as any evidence that a patient had had syphilis.

Dr. GOULD asked for an explanation of Dr. Cronyn's remark as to the occurrance of hemmorrhage after the use of Esmarch's bandage.

Dr. CRONYN said that the bandage expelled the blood completely from the capillaries. Their contractility seemed to be impaired by the severe pressure, and upon removal of the bandage, these vessels would bleed profusely. He had seen excessive hemmorrhage occur in this way. The best demonstration of this could be seen in a primary amputation of the thigh, in a person in full vigor. In secondary operations there was less liability to hemmorrhage.

Dr. MINER cited a case of amputation at the hip joint for caries, reported at a recent meeting, in which he had used Esmarch's apparatus. The tissues were divided at the same point as in amputating at the upper third of the thigh, and the bone was disarticulated after the method of Dr. McGraw, of Detroit. The only embarrasment caused by the apparatus was the difficulty of finding the vessels to be tied. No hemmorrhage succeeded the application. He had however seen one case in which removal of the bandage had been followed by pretty free hemmorrhage.

With regard to the use of the galvano-cautery, many operations could be performed as well or better by other means, but there were some cases for which it might be peculiarly adapted, as in cauliflower excrescence of the cervix uteri.

Dr. LYNDE had used Esmarch's bandage in a case of caries of the bones of the foot. The tissues were much congested, and he did not succeed in fully expelling the blood, so that bleeding attended the operation. The removal of the bandage was followed by a gush of blood, but not excessive. He did not think the use of the bandage objectionable in operations below the knee, but where a large surface was compressed as in amputation of the thigh, dangerous hemmorrhage might ensue.

Dr. Barnes wished to say in reference to the assertion of Dr. Cronyn, that when the wire loop was used with the battery, it acted simply as an écraseur; that while he would not deny the accuracy of the gentleman's observations in Europe or elsewhere, yet in the case which he had reported, the loop did not act as an écraseur in any degree. The wire, when heated to a white heat, is incapable of sustaining much tension. If it is not kept wholly in contact with the tissues, so that its heat may be constantly abstracted, the exposed portion is liable to fuse or to part upon very gentle traction, an accident he had seen occur more than once. In this case, the loop was adjusted about the tumor and tightened so as firmly to compress the tissues, but not to penetrate their substance. Then the circuit was completed, the wire was instantly heated, and without further traction buried itself deeply, with a hissing sound, until the limit of expansibility of the compressed tissues was reached. A few turns of the screw, with scarcely appreciable resistance, completed the operation. The time occupied seemed hardly more than four seconds.

Dr. Lynde reported a case of hydrothorax in which there was a spontaneous yielding or rupture of the pleural membrane, followed by infiltration of the areolar tissue on the corresponding side, from the hip to the shoulder. He had never seen nor read of a similar case. He also reported the case of an infant exhibiting an arrest of development of abdominal muscles, and of a portion of the anterior chest walls. The heart was in the median line covered only by a thin membrane, so that it was distinctly visible and palpable. It might almost be circumscribed by the fingers and lifted from its position.

On motion, the Society adjourned.

Note.—The patient operated on by the galvano cautery, progressed rapidly and favorably. The slough which was quite superficial separated in a few days leaving a healthy sore, A tendency to a recurrence of the growths subsequently manifesting itself, chromic acid was freely applied, producing a deep slough, and finally eradicating the vegetations.

